# Supplementary material for: Correlative ultrastructural mapping of Lewy pathology reveals regional diversity in Parkinson’s and dementia with Lewy bodies
Source: Acta Neuropathol. 2026 Jul 8;152(1):5. doi: 10.1007/s00401-026-03039-w (PMC13346217; doi:10.1007/s00401-026-03039-w)
Supplement: Supplementary file 1 — Supplementary file1 (PDF 2695 KB) [file 401_2026_3039_MOESM1_ESM.pdf]

Supplementary Information

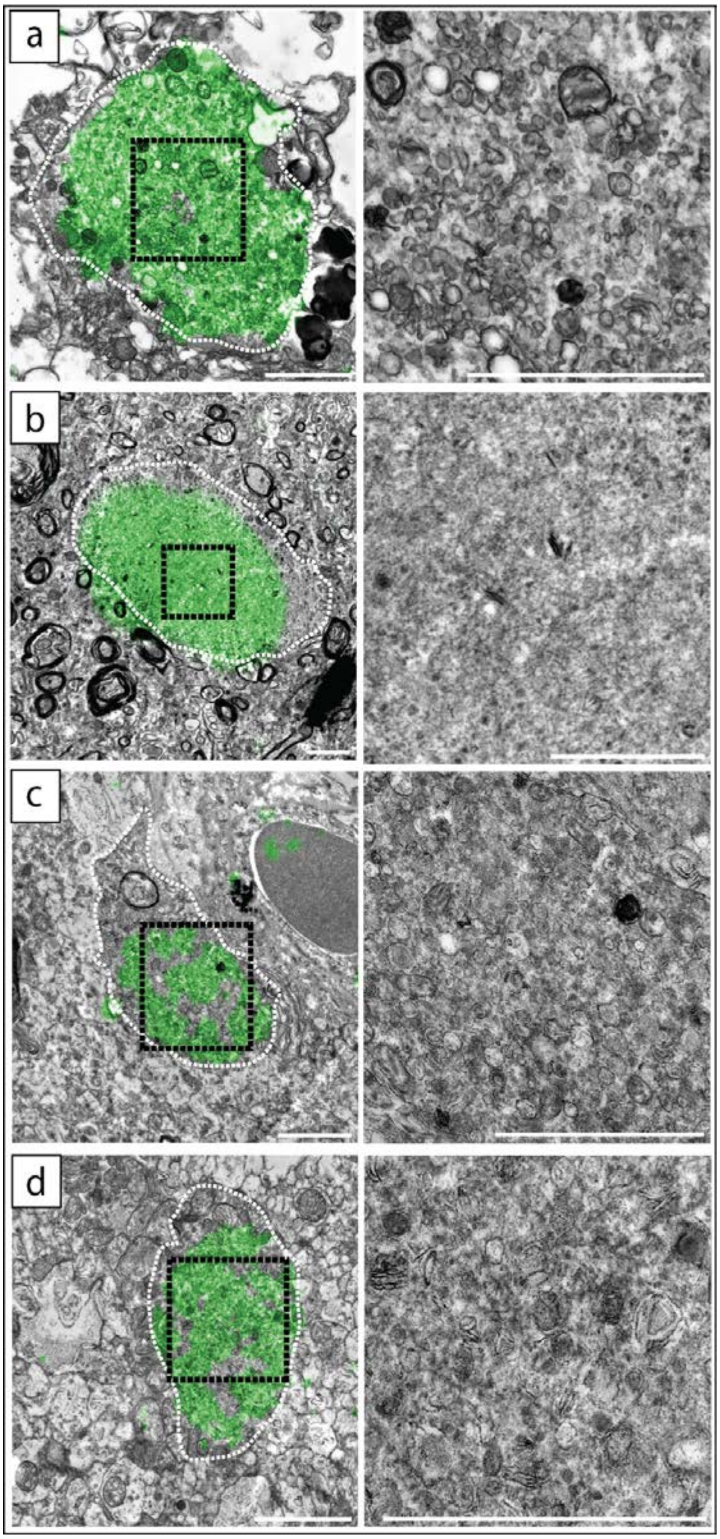

Supplementary Figure 1. Neuritic pathology in ACC of DLB and PD cases. In all panels, dashed lines outline neuritic boundaries; black boxed areas indicate higher magnification views. a) Membranous neuritic inclusion in the ACC of DLB case. b) Fully fibrillar neuritic inclusion in ACC of DLB case. c, d) Membranous neuritic inclusion in the ACC of PD cases. The IHC α-Syn immunostaining (green) is overlaid onto the EM micrograph. Scale bars: 2 μm.

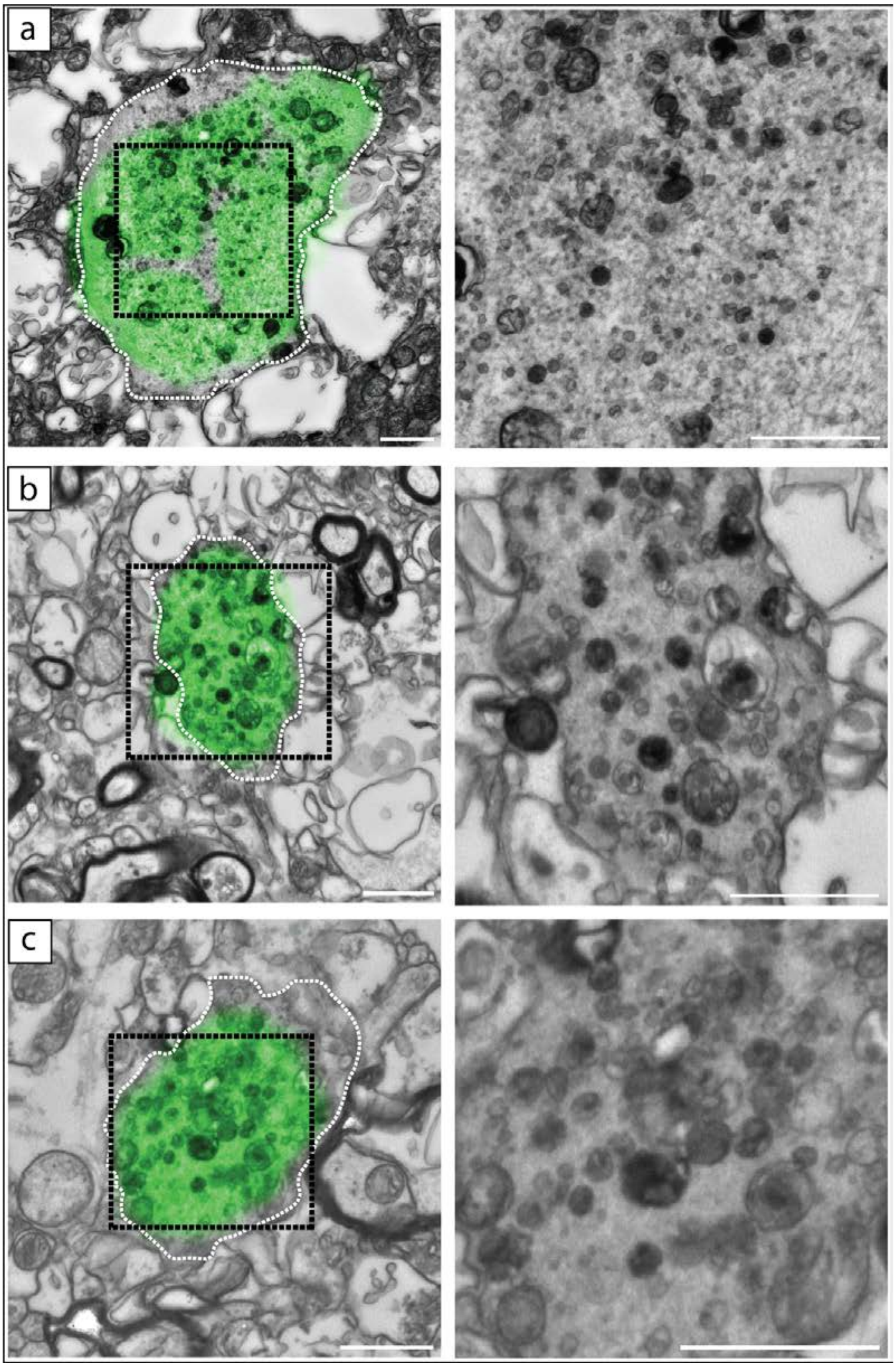

Supplementary Figure 2. Neuritic pathology in ENT and CA2 of PD cases. In all panels, dashed lines outline neuritic boundaries; black boxed areas indicate higher magnification views. a) Mixture of membranes and fibrils neuritic inclusion in the ENT of PD case. b) Mixture of membranes and fibrils neuritic inclusion in CA2 of PD case. c) Another example of mixture of membranes and fibrils neuritic inclusion in CA2 of PD case The IHC α-Syn immunostaining (green) is overlaid onto the EM micrograph. Scale bars: 1 µm.

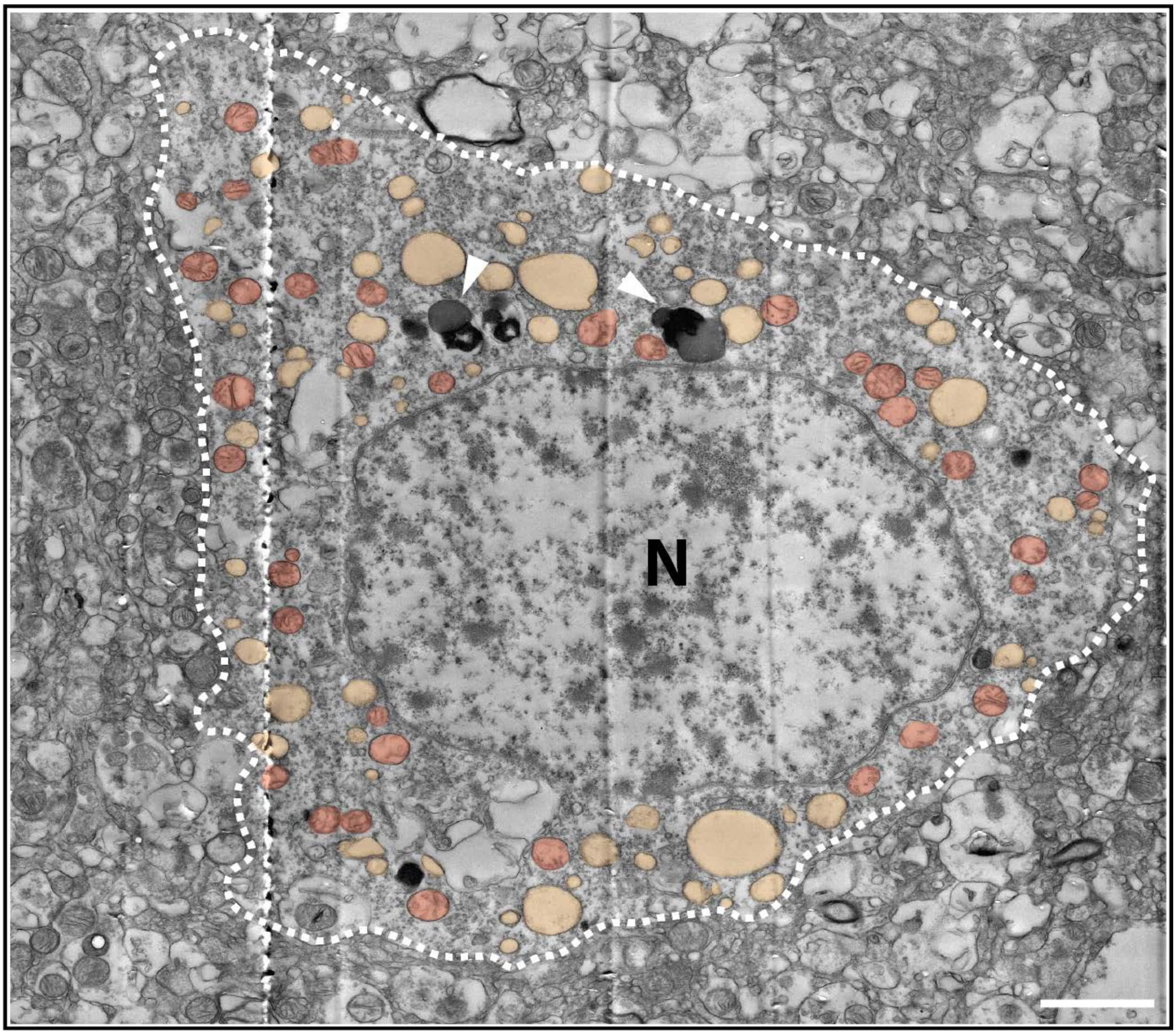

Supplementary Figure 3. Neuron from the ENT of a non-neurological control. N refers to the nucleus, and the membrane is indicated by the white dashed line. Mitochondria (red), other vesicles and organelles (yellow) are annotated. The nucleus is in the middle of the neuron, some lipofuscin droplets can be seen (white arrows), and there is no empty space between the neuron and the surrounding tissue. Scale bars: 2 µm

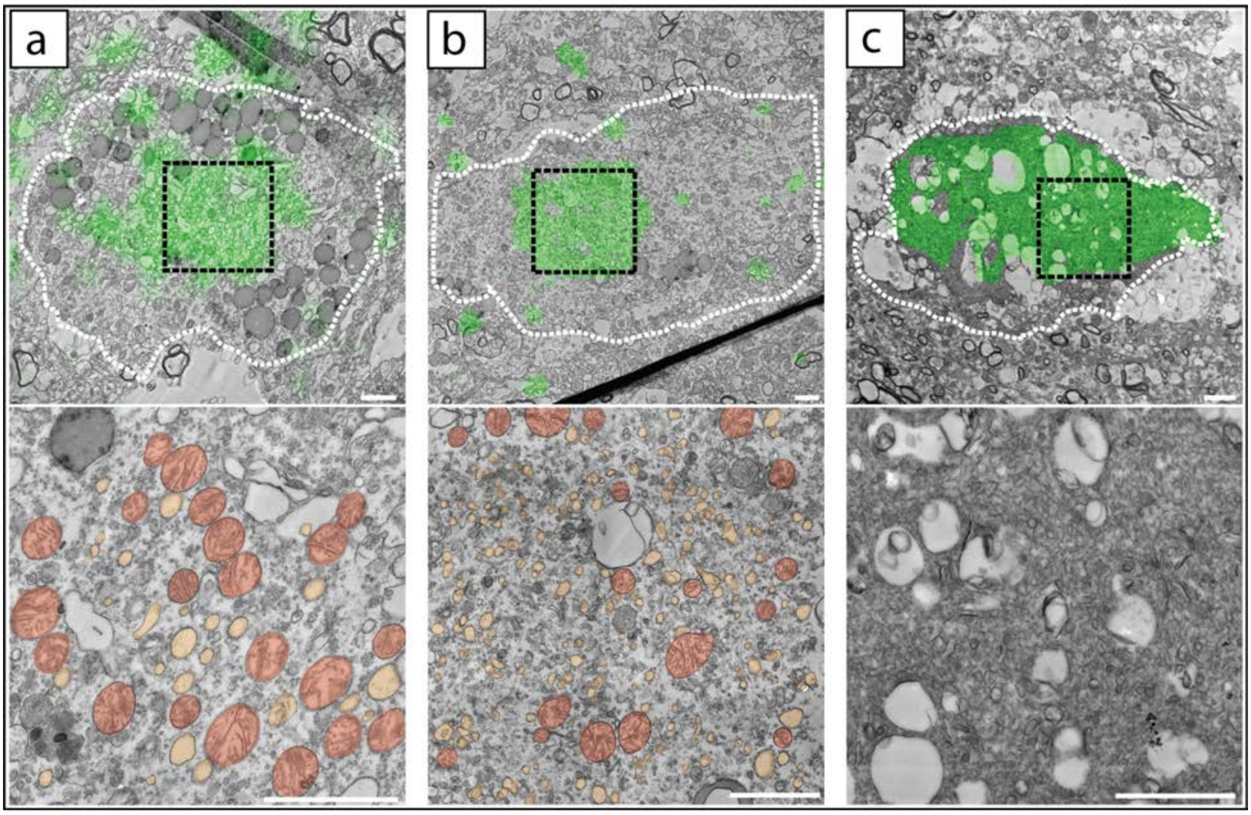

Supplementary Figure 4. Irregular ultrastructures observed in the CA2 of DLB cases. In all panels, dashed lines outline neuritic boundaries; black boxed areas indicate higher magnification views. Mitochondria (red), other vesicles and organelles (yellow) are annotated. a) The somatic ultrastructure in CA2, there are low density fibrils mixed with other organelles, b) Another example of somatic LB in CA2 with the ultrastructure as A. c) Irregular p-α-Syn positive region no fibrils have been observed. The IHC α-Syn immunostaining (green) is overlaid onto the EM micrograph. Scale bars: 2 μm.

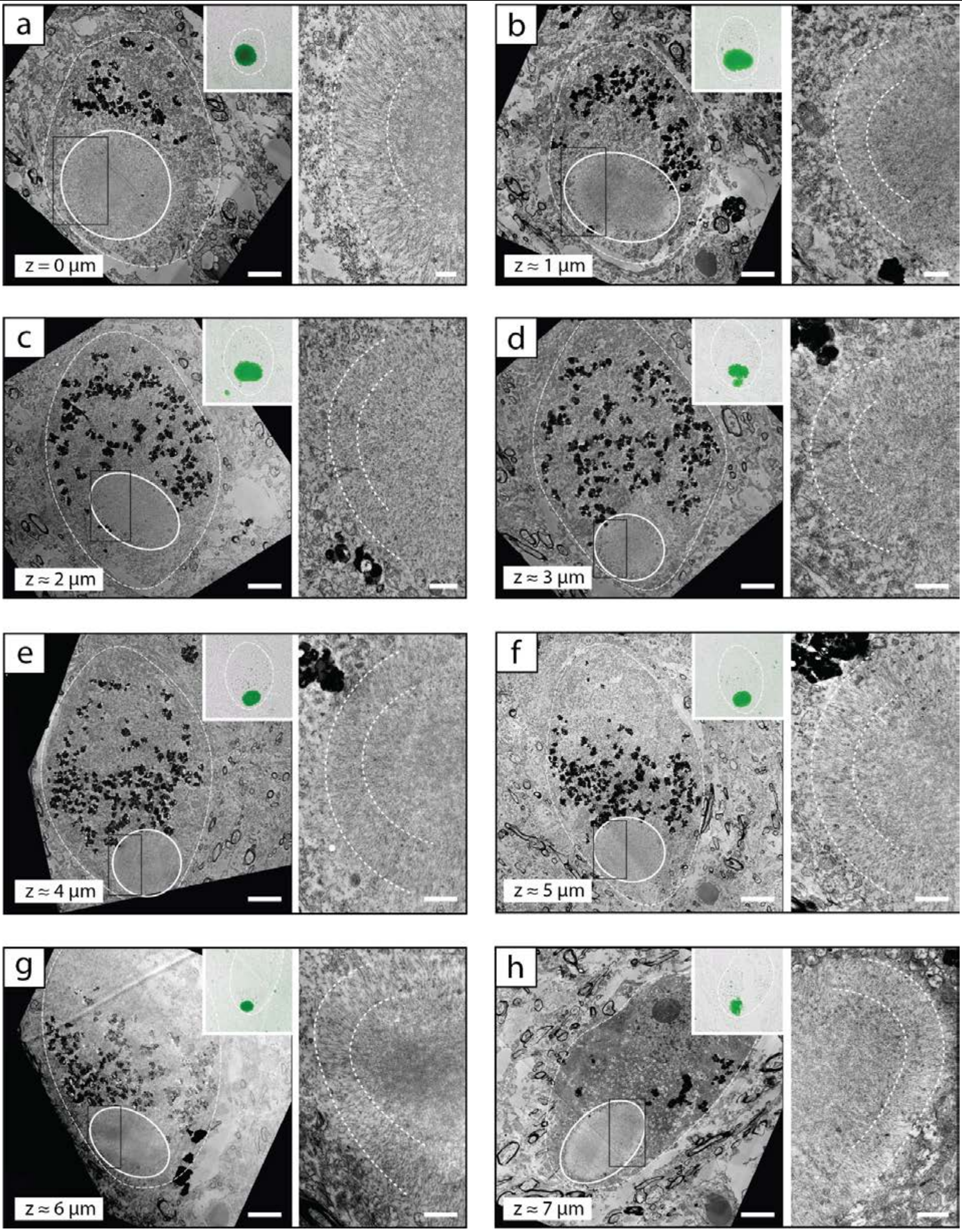

Supplementary Figure 5: Serial section analysis of a 2-layer nigral LB. The same LB is shown across eight consecutive sections (~1 µm apart) with each panel representing a different z-plane through the volume. This LB corresponds to Fig. 3C, with panel F identical to the main figure panel shown again here for completion. Across all sections, the LB consistently shows a 2-layered organization, with no evidence of an additional electron dense core, supporting that 2-layer LBs are not sectioning artefacts of 3-layer LB. Insets show α-Syn immunohistochemistry. Scale bars (low mag): 5 µm, others 1 µm.

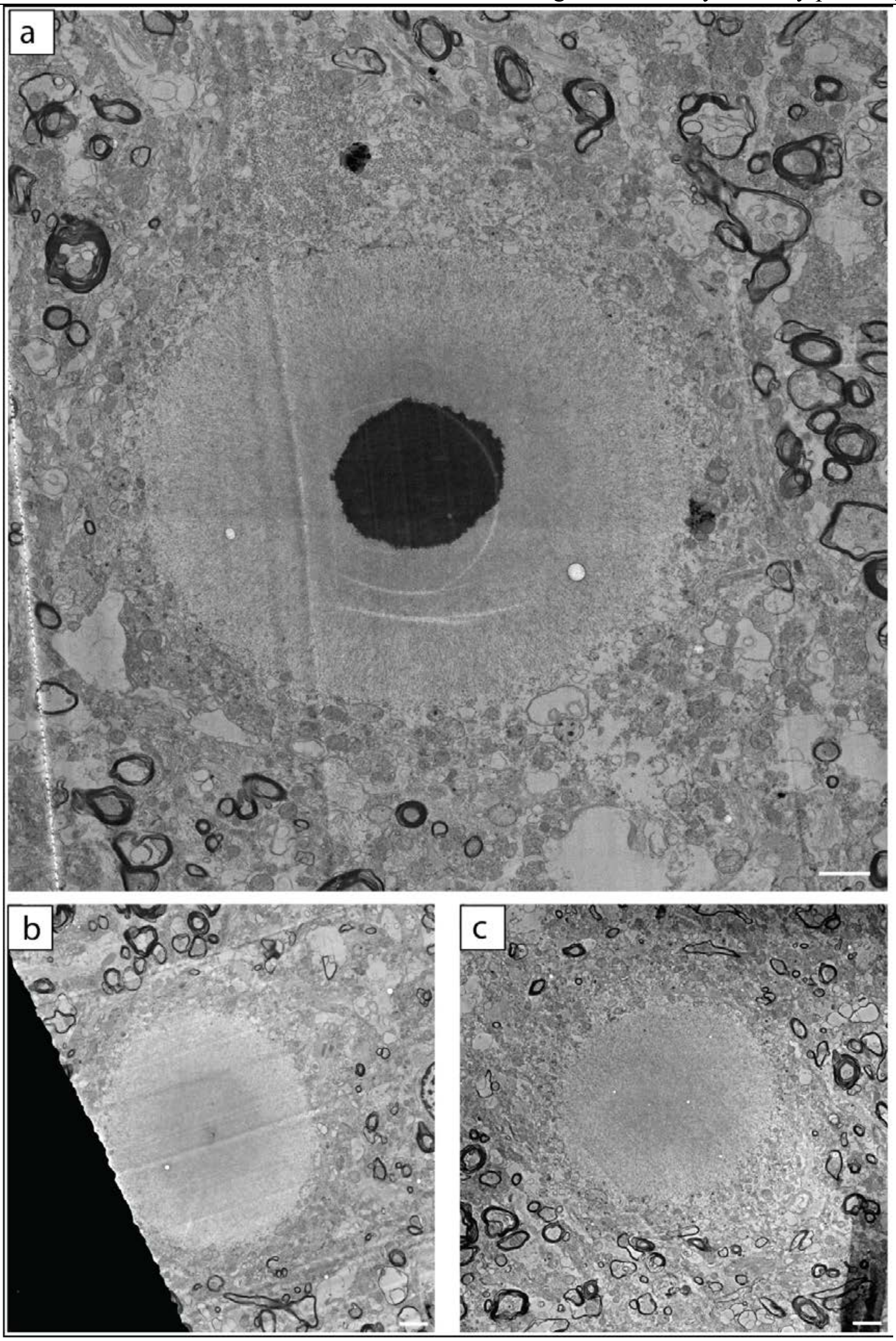

Supplementary Figure 6. Serial section analysis of a three-layer LB in SN of DLB case. a) Shows the three–layer LB in the first cycle. b) Shows the three–layer LB in third cycle. c) Shows the three–layer LB in the fourth cycle. The image shown in the main figure (Figure 3) obtained from cycle 2. Scale bars: 2 μm.

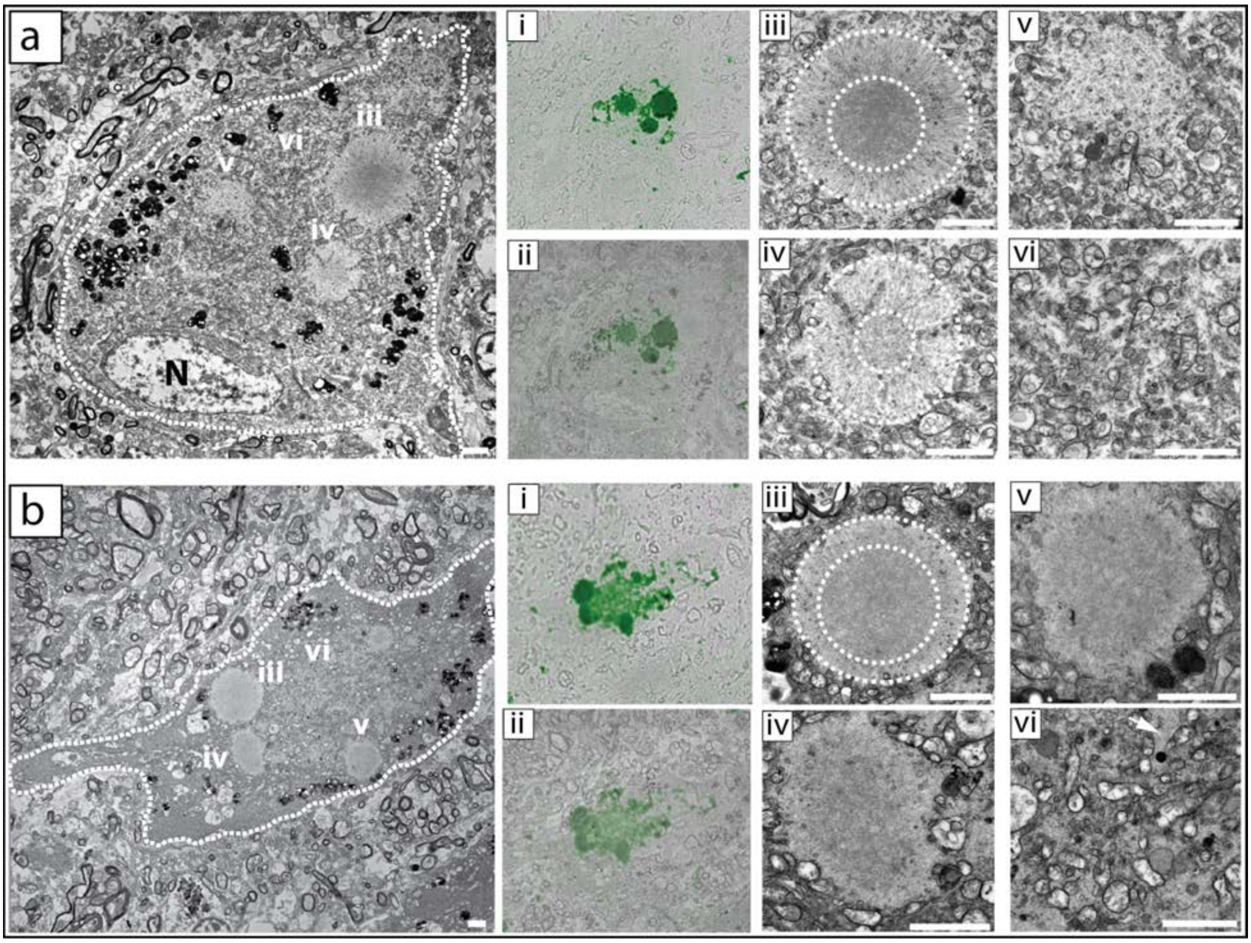

Supplementary Figure 7. Examples of Multiple LBs in one neuron in SN of DLB cases. a) Shows an example of multiple LB in one neuron which are connected by mitochondria. i) show α-Syn immunohistochemistry of the region, ii) shows the overlay of the EM and immunohistochemistry, iii) shows two-layer LB ultrastructure, iv) shows another two-layer LB as the second baby LB, v) shows one-layer LB which has no arranged fibrils, vi) show the area between these pathology which contains packed mitochondria with a few fibrils. b) Shows another example of multiple LBs in one neuron. i) show α-Syn immunohistochemistry of the region, ii) shows the overlay of the EM and immunohistochemistry, iii) shows two-layer LB ultrastructure, iv) shows one-layer LB, v) shows another one-layer LB, vi) show the pale body which connects LBs. Scale bars: 2 µm.

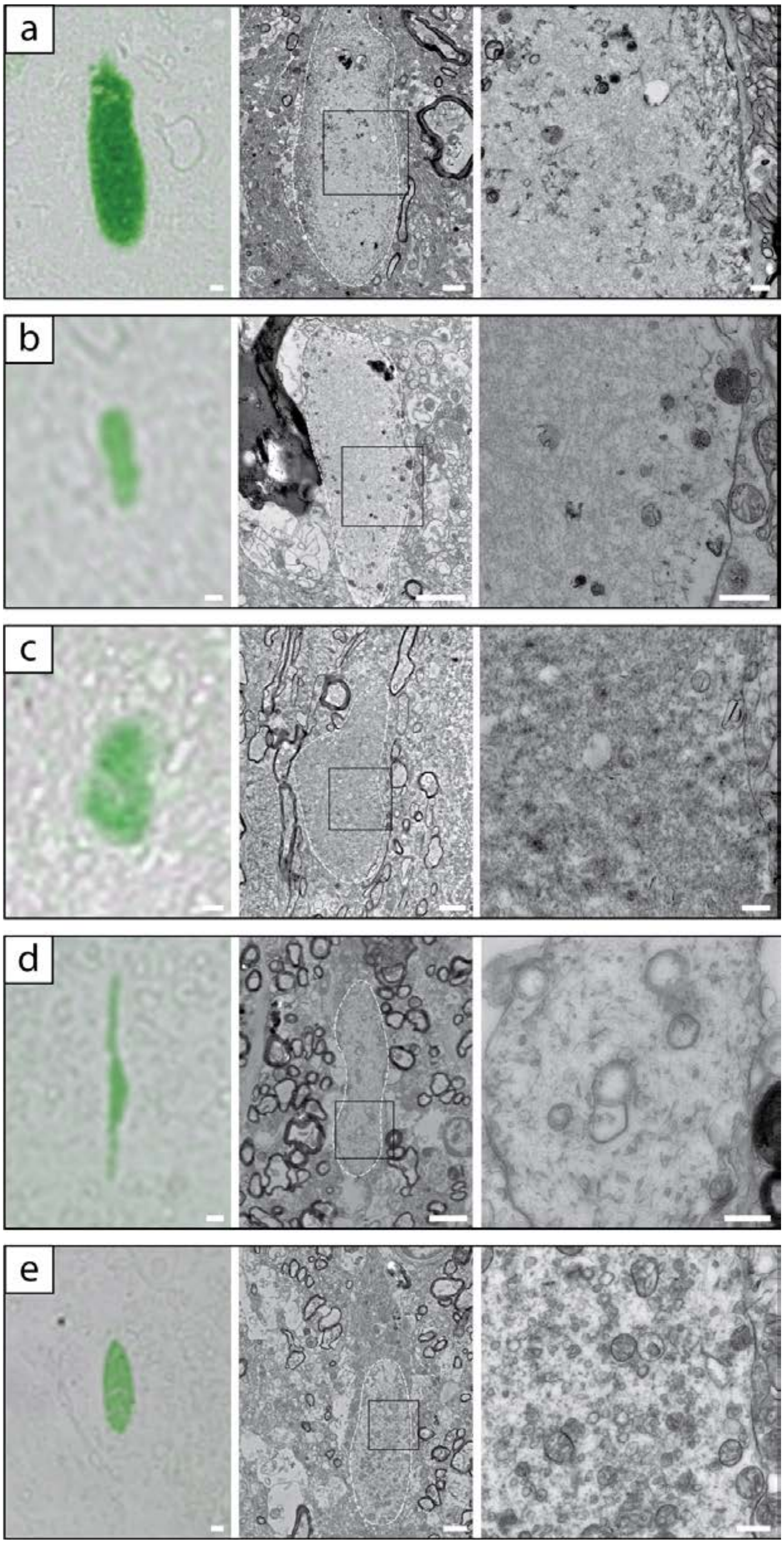

Supplementary Figure 8: Examples of Lewy neurites in the SN from DLB donors sectioned along their longitudinal axis. The first column shows the α-Syn immunohistochemistry, correlated EM micrographs shown in the middle column. Higher magnification areas (block box) shown in the last column. Scale bars (low mag): 2 µm, others 0.5 µm.

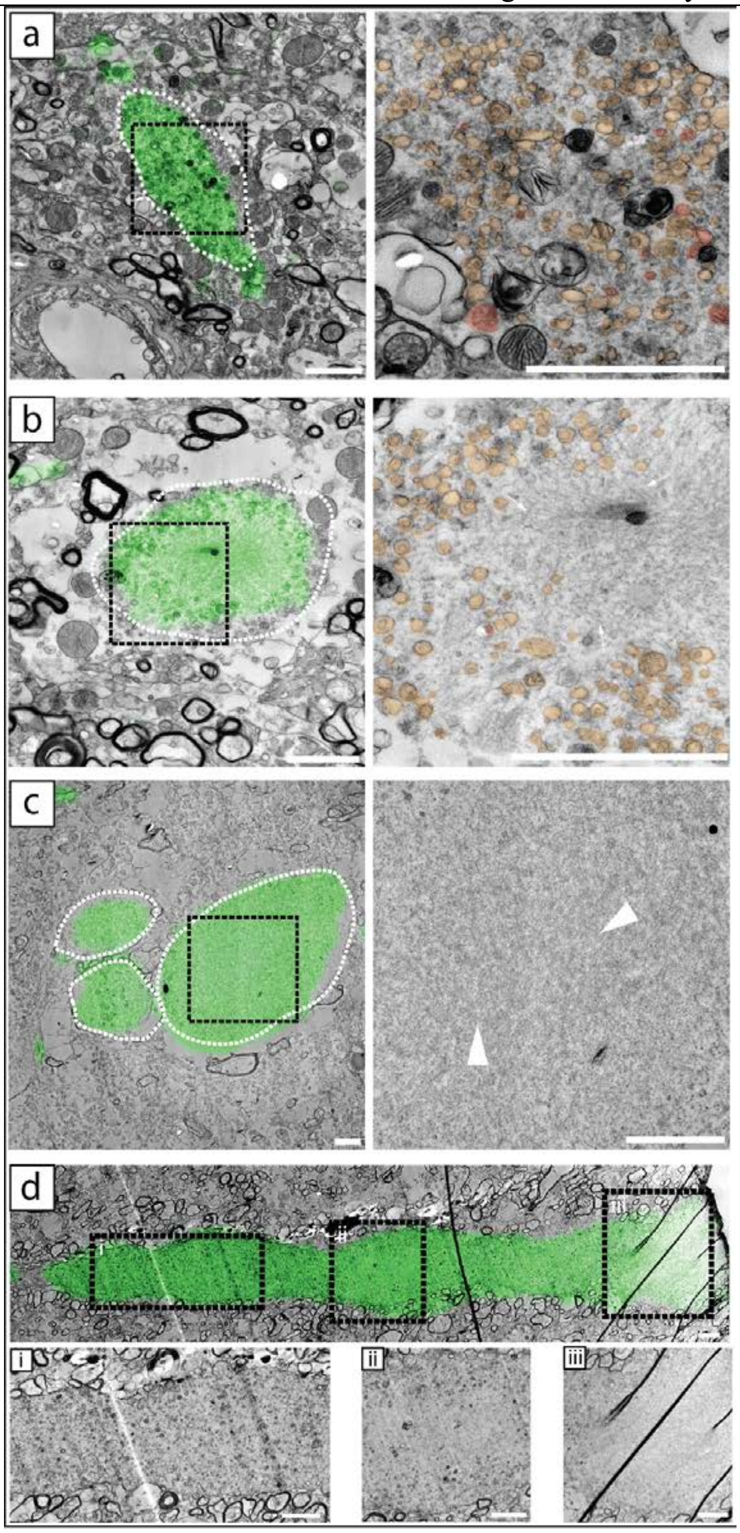

Supplementary Figure 9. Neuritic pathology in CA2 in DLB cases. In all panels, dashed lines outline neuritic boundaries; black boxed areas indicate higher magnification views. Mitochondria (red), other vesicles and organelles (yellow) and regions of fibrils (white arrowheads) are annotated. a) Membranous neuritic inclusion in the CA2. b) Central fibrillar and surrounded by other organelles. c) Fully fibrillar non somatic. d) Bulky neurite around 60 µm in length from the CA2 (top original image indicating cut-outs, bottom with IHC overlaid). Cutouts in i) show mixed fibrillar and other organelles, ii) more fibrillar, and iii) fully fibrillar but not positive for p-α-Syn. The IHC α-Syn immunostaining (green) is overlaid onto the EM micrograph. Scale bars: 2 µm.

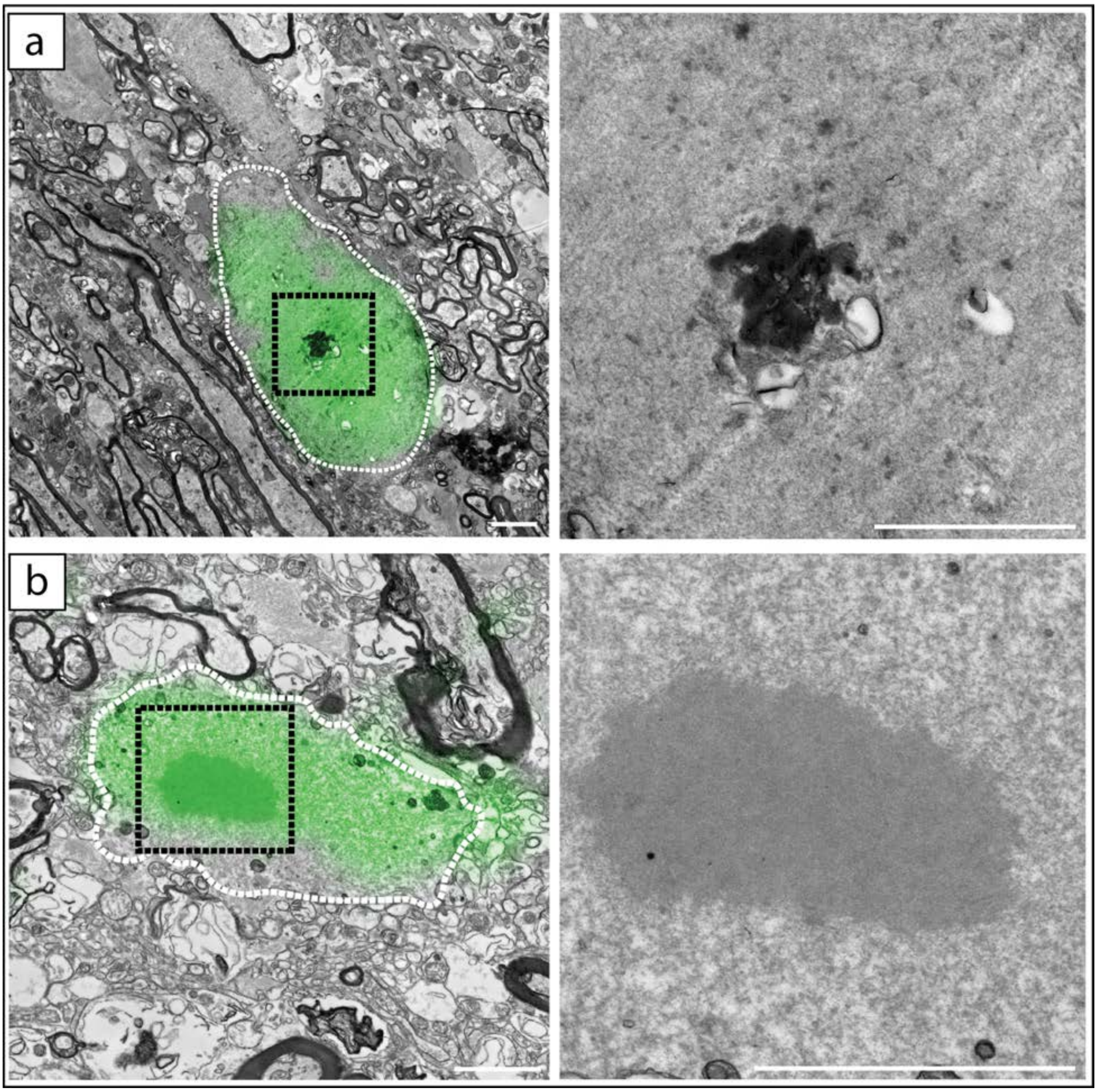

Supplementary Figure 10. Other examples of neuritic pathology with electron dense core in SN of DLB cases. In all panels, dashed lines outline neuritic boundaries; black boxed areas indicate higher magnification views. a) shows the example of fibrillar neuritic pathology with a dark core. b) Shows another example with lower electron core density**.** The IHC α-Syn immunostaining (green) is overlaid onto the EM micrograph. Scale bars: 2 μm.

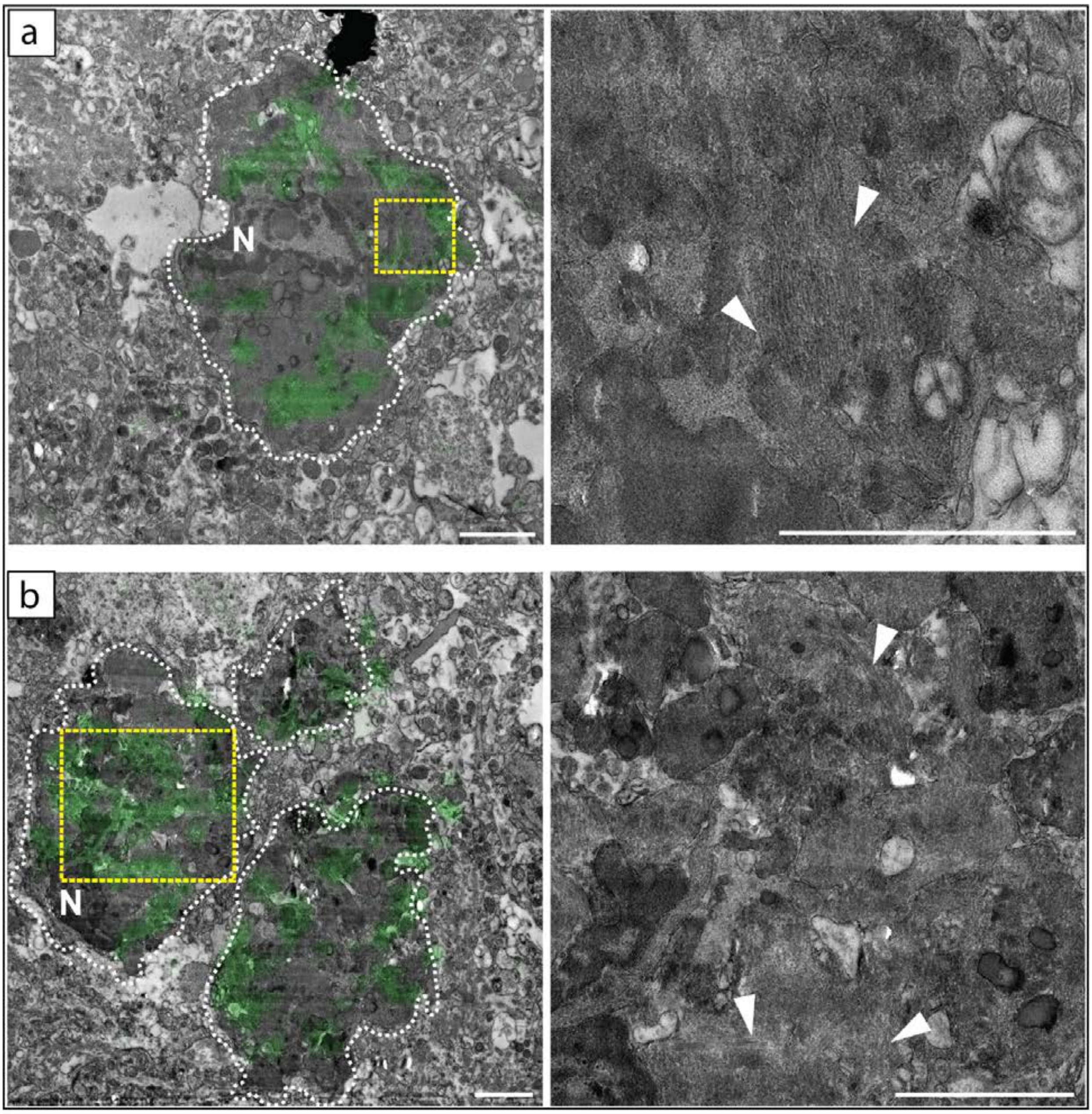

Supplementary Figure 11. Irregular pathology in ENT from DLB case. In all panels, dashed lines outline neuritic boundaries; yellow boxed areas indicate higher magnification views. Regions of fibrils (white arrowheads) are annotated. a) Degenerating neuron positive for p-α-Syn in the ENT; the nucleus appears dark. b) Somatic and non-somatic α-Syn-positive region associated with the degenerating neuron. Dense packed oriented fibrils are indicated by white arrow. The IHC α-Syn immunostaining (green) is overlaid onto the EM micrograph. Scale bars: 2 µm.

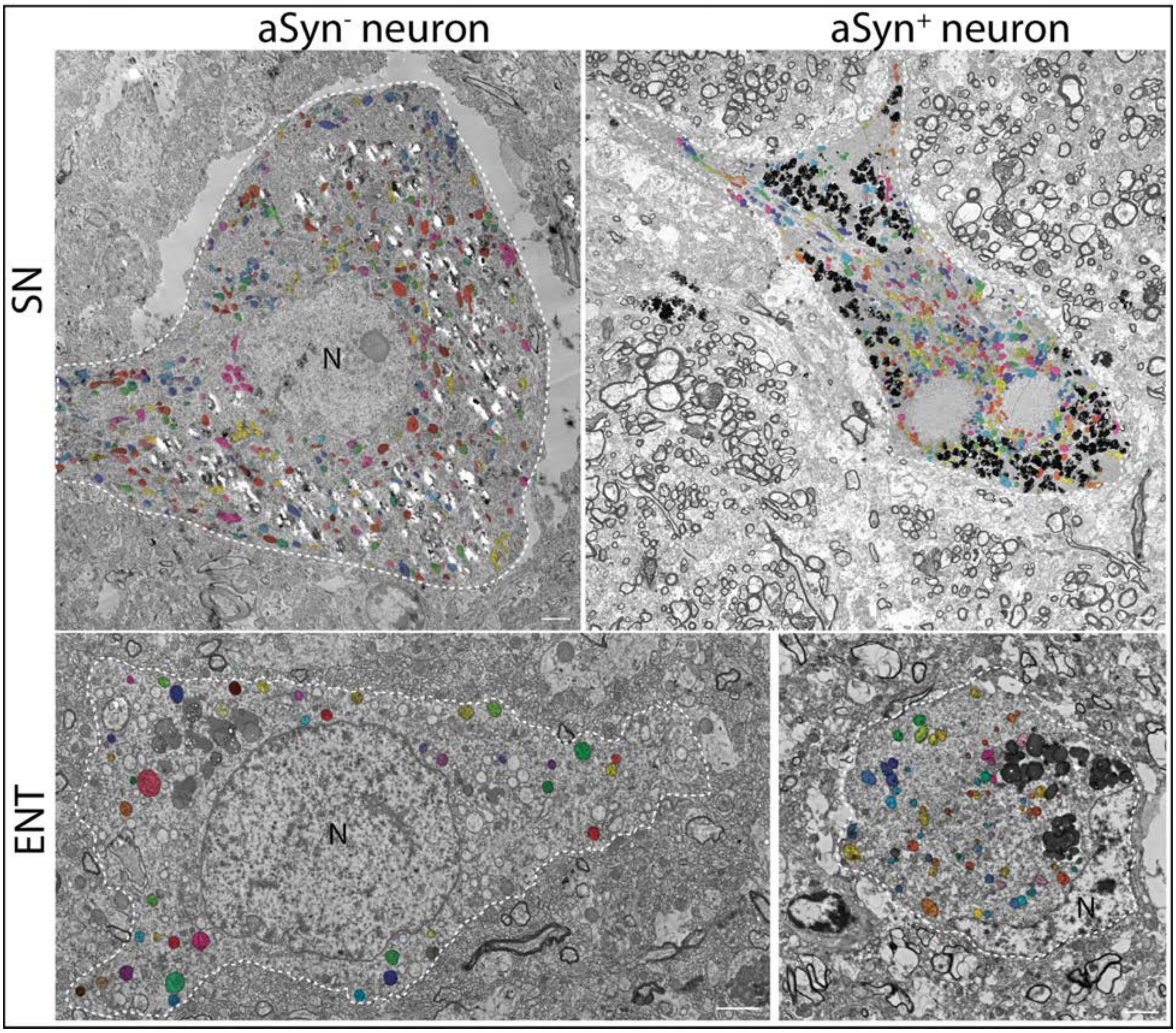

Supplementary Figure 12. Segmented mitochondria in representative cells from DLB donors. The white dashed line indicates the cell boundary, examples of segmented mitochondria are coloured, the nucleus is indicated where visible (N). Scale bars 2 µm.

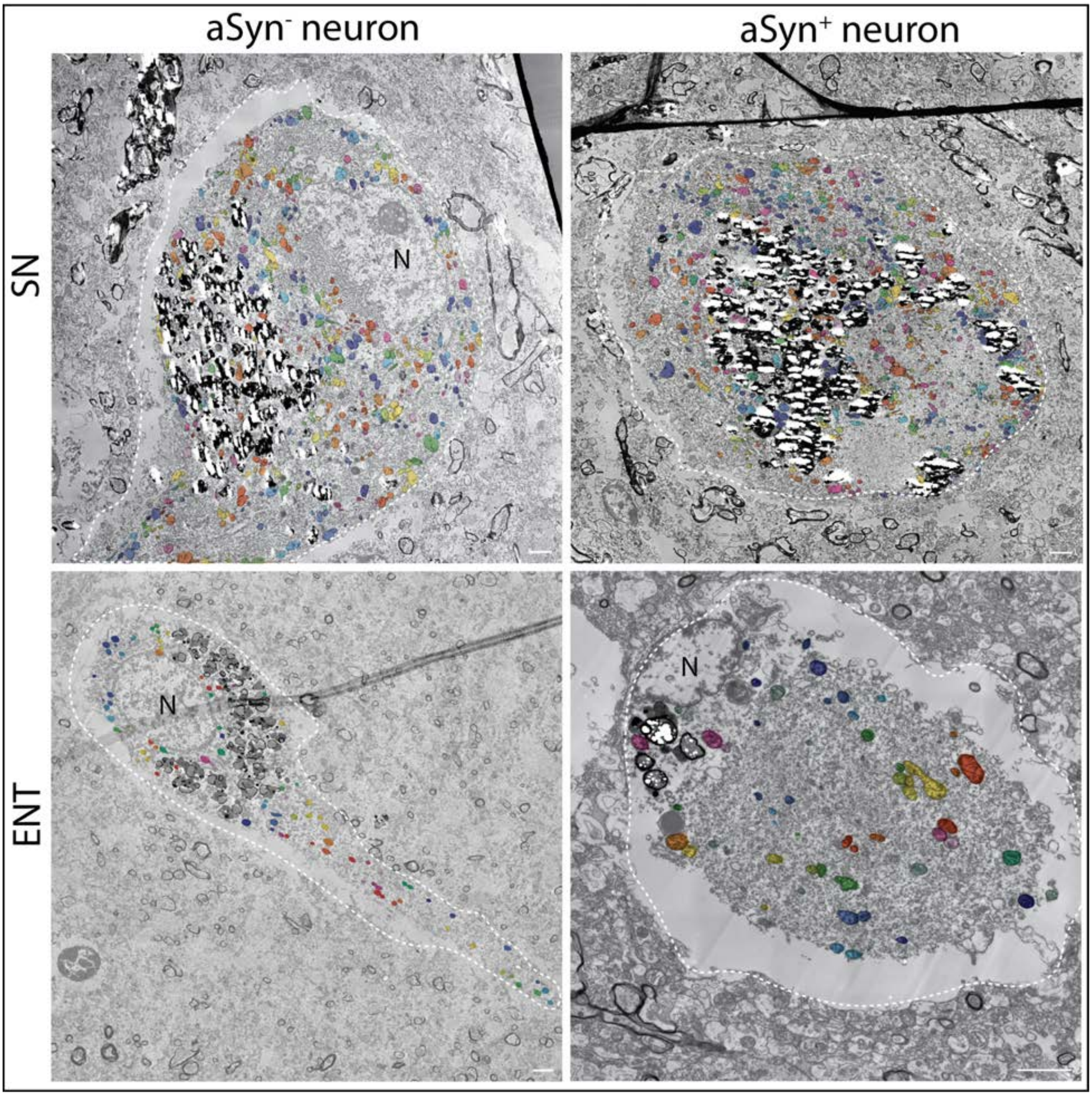

Supplementary Figure 13. Segmented mitochondria in representative cells from PD donors. The white dashed line indicates the cell boundary, examples of segmented mitochondria are coloured, the nucleus is indicated where visible (N). Scale bars 2 µm.

Case	F/M	Age at death (y)	Diagnosis		PMD (h:m)	Brain weight (g)	Braak α-Syn stage	Braak NFT	Thal phase
			Clin.	Path.					
DLB 1	M	72	DLB	DLB+AD	04:15	1397	6	3	4
DLB 2	M	71	DLB	DLB	04:15	1400	6	2	2
DLB 3	M	68	DLB	DLB	04:55	1605	6	2	3
DLB 4	F	77	DLB	DLB	06:45	1185	6	2	3
PD 1	M	88	PDD	PDD	4:05	1405	6	3	3
PD 2	M	82	PDD	PDD	6:50	1200	6	2	0
PD 3	M	83	PD	PD	6:25	1387	6	3	1
PD 4	M	74	PD	PDD	5:15	1350	6	2	3
PD 5	M	84	PD	PD	4:50	1430	5	3	0
Cont 1	F	74	Cont	Cont	6:10	n.d	1	2	3
Cont 2	M	63	Cont	Cont	3:00	1350	0	1	1

Supplementary Table 1. Clinicopathological features of donors included in this study. DLB=Dementia with Lewy bodies; PD=Parkinson's disease; AD=Alzheimer disease; Cont=control; F/M=female/male; y=year; Clin.=clinical diagnosis; Path.=pathological diagnosis; PMD= postmortem delay; h=hours; m=minutes; g=grams;NFT=neurofibrillary tangle. n.d= no data available.

Disease	Brain region	Fibrillar density			Total # inclusions	
		Low density	Mid density	High density		
Cortical LBs					Per brain region	Per disease
DLB	ENT	20	20	2	42	66
	ACC	n.o.	n.o.	20	20	
	CA2	2	n.o.	n.o.	2	
PD	ENT	9	4	3	16	26
	ACC	n.o.	n.o.	10	10	
Degenerating neurons						
DLB	ENT	2	3	n.o.	5	7
	ACC	n.o.	0	2	2	
PD	ACC	n.o.	n.o.	1	1	1
Other brain regions						
DLB	SN				72	72
Neuritic pathology						
DLB	ACC				2	64
	CA2				6	
	ENT				13	
	SN				43	
SN	ENT				2	7
	CA2				5	
Irregular ultrastructure						
DLB	ENT				10	11
	CA2				1	

Supplementary Table 2. Classification and count of α-Syn pathology localized by CLEM. DLB=Dementia with Lewy bodies; PD=Parkinson's disease; ENT=entorhinal cortex; ACC= anterior cingulate cortex; CA2=hippocampus; SN=substantia nigra; n.o. = not observed.
